# Morphological, Behavioral, and Transcriptomic Profiling Reveals Developmental Toxicity of PCB Metabolites in Zebrafish

**DOI:** 10.3390/toxics14050444

**Published:** 2026-05-19

**Authors:** Nicole M. Breese, Lisa Truong, Xueshu Li, Robyn L. Tanguay, Hans-Joachim Lehmler

**Affiliations:** 1 Department of Occupational and Environmental Health, College of Public Health, The University of Iowa, Iowa City, IA 52242, USA; nicole-breese@uiowa.edu (N.M.B.); xueshu-li@uiowa.edu (X.L.); 2 Interdisciplinary Graduate Program in Human Toxicology, The University of Iowa, Iowa City, IA 52242, USA; 3 Sinnhuber Aquatic Research Laboratory, Department of Environmental and Molecular Toxicology, Oregon State University, Corvallis, OR 97331, USA; lisa.truong@oregonstate.edu (L.T.); robyn.tanguay@oregonstate.edu (R.L.T.)

**Keywords:** metabolites, mixtures, neurotoxicity, polychlorinated biphenyls, transcriptomics, zebrafish

## Abstract

Polychlorinated biphenyls (PCBs) persist in the environment as complex mixtures and undergo extensive biotransformation, yet the developmental toxicity of PCB metabolites remains poorly defined. We evaluated developmental, neurobehavioral, and molecular effects of parent PCBs, hydroxylated, methoxylated, and sulfated metabolites, and environmentally relevant mixtures using embryonic zebrafish. This study employed a high-throughput screening approach using nominal exposure concentrations to enable rapid hazard identification and prioritization across a large chemical series. Morphological abnormalities and photomotor behavior were assessed across early development, followed by targeted *cyp1a* reporter analysis and transcriptomic profiling for a subset of potent exposures. Most chemicals induced morphological effects, with hydroxylated and sulfated metabolites producing effects more frequently and at lower benchmark concentrations than parent congeners. Behavioral alterations were more prevalent in embryonic photomotor response than larval photomotor response and generally co-occurred with morphological effects. Environmental mixtures elicited broad phenotypic profiles comparable to highly active individual compounds. Transcriptomic analyses revealed minimal responses for parent PCBs but robust, exposure-specific gene expression changes for select metabolites, particularly 5-OH-PCB11, and mixtures. Differentially expressed genes were enriched for xenobiotic metabolism, immune signaling, and neuroactive pathways, alongside consistent downregulation of circadian regulators. Together, these results demonstrate contributions of PCB metabolites and mixtures to toxicity.

## 1. Introduction

Polychlorinated biphenyls (PCBs) are a class of chlorinated aromatic hydrocarbons that remain persistent global contaminants decades after regulatory bans [1]. This is due to their chemical stability, resistance to environmental degradation, and extensive historical use in industrial and commercial applications [1,2,3]. Although production of technical PCB mixtures was banned or severely restricted in many countries beginning in the 1970s, PCBs remain globally distributed in soils, sediments, and building materials [4,5]. Human exposure to PCBs arises from legacy contamination [6,7], bioaccumulation through food webs [8], and ongoing inadvertent production of non-legacy congeners such as PCB11 during pigment and chemical manufacturing [6,7]. Epidemiologic and experimental studies have linked PCB exposure to adverse neurodevelopmental outcomes, including impaired cognitive function, behavioral alterations, and developmental delays [9,10]. In addition to developmental effects, PCB exposure is associated with cognitive dysfunction across the lifespan and may contribute to risk for neurodegenerative diseases [11,12].

PCB congeners are often categorized into functional classes based on structure and mechanism. Dioxin-like PCBs (DL-PCBs) exert toxicity primarily through activation of the aryl hydrocarbon receptor (AhR) and are assessed using toxic equivalency approaches [13]. However, many congeners associated with neurodevelopmental toxicity are non-dioxin-like PCBs (NDL-PCBs) that exhibit no or weak AhR activity, yet produce behavioral and neurodevelopmental effects [14]. These congeners act through AhR-independent mechanisms, including disruption of intracellular calcium signaling, altered neurotransmitter systems, and oxidative stress pathways [14]. Importantly, real-world PCB exposures involve complex mixtures of congeners rather than individual compounds [15,16,17].

PCBs undergo extensive biotransformation through Phase I oxidation and Phase II conjugation, producing hydroxylated, methoxylated, and sulfated PCB metabolites [18]. Hydroxylated PCBs (OH-PCBs) and PCB sulfates are consistently detected in human serum [19,20], yet the toxicology of PCB metabolites remains less characterized than that of parent congeners. Certain metabolites alter neuronal calcium signaling by sensitizing ryanodine receptors, a pathway critical for activity-dependent neurodevelopment, and in some systems display equal or greater potency than their parent compounds [21,22,23]. These findings indicate that PCB metabolites may contribute to neurodevelopmental toxicity through mechanisms distinct from classical dioxin-like pathways [10,14,24].

PCB toxicology is heterogeneous, as congeners and their metabolites differ in chlorination patterns, physicochemical properties, and metabolic fate, influencing tissue distribution and molecular interactions [18,25]. Toxic outcomes emerge from multidimensional interactions across chemical structure, organismal behavior, tissue morphology, and gene expression [26]. Systems-level approaches that integrate diverse biological endpoints are needed to identify patterns not evident in single-domain analyses [27]. Multi-endpoint profiling frameworks such as the Toxicological Priority Index (ToxPi) were designed for objective chemical prioritization by formally integrating heterogeneous data across biological domains into a transparent, weighted scoring system [28]. Here, morphological, behavioral, and transcriptomic profiles from zebrafish embryos were incorporated to facilitate the classification of PCB congeners, metabolites, and mixtures based on cumulative bioactivity [29].

Despite extensive studies of PCB toxicity, key uncertainties remain. It is unclear whether PCB metabolites are more potent developmental neurotoxicants than their parent congeners, whether functional behavioral endpoints provide greater sensitivity than morphology alone, and to what extent developmental neurotoxicity is mediated through AhR-dependent versus AhR-independent mechanisms in vivo. It is also unknown whether structurally diverse PCBs alter shared transcriptional programs during development or act through distinct molecular pathways, and whether integrated multi-domain profiling can identify mechanistic subclasses beyond traditional congener-based classifications.

Zebrafish (*Danio rerio*) are a powerful vertebrate model for developmental neurotoxicity because it combines conserved neurodevelopmental processes with experimental features that enable simultaneous structural, functional, and molecular assessment [30,31]. Key neurodevelopmental events occur rapidly and in patterns paralleling mammalian systems, while embryonic transparency permits in vivo visualization of morphology and neuroanatomy [32,33,34]. Zebrafish are also well suited for high-throughput behavioral screening, allowing quantitative evaluation of neurodevelopmental function across large numbers of individuals under controlled exposure paradigms [35]. Importantly, zebrafish assays are readily integrated with transcriptomic approaches, enabling the connection of behavioral phenotypes with molecular pathway alterations [35].

To address these gaps, we systematically evaluated the developmental and neurobehavioral toxicity of parent PCBs, hydroxylated, methoxylated, and sulfated metabolites, and environmentally relevant mixtures using a zebrafish model. This study was designed as a high-throughput screening approach encompassing a large series of PCBs and related compounds, where nominal exposure concentrations are used to enable rapid hazard identification and prioritization rather than detailed toxicokinetic characterization. High-throughput morphological screening was combined with embryonic and larval behavioral assays to capture structural and functional neurodevelopmental outcomes. A transgenic *cyp1a* reporter line quantified AhR-pathway activation, while transcriptomic profiling at a pre-morphological time point identified early molecular alterations preceding overt phenotypes. This data was integrated using ToxPi–based multi-domain analysis to determine how PCB structure and metabolic transformation relate to biological activity and to identify shared and divergent mechanisms underlying early-life PCB toxicity.

## 2. Materials and Methods

### 2.1. Chemicals

**Figure 1:**
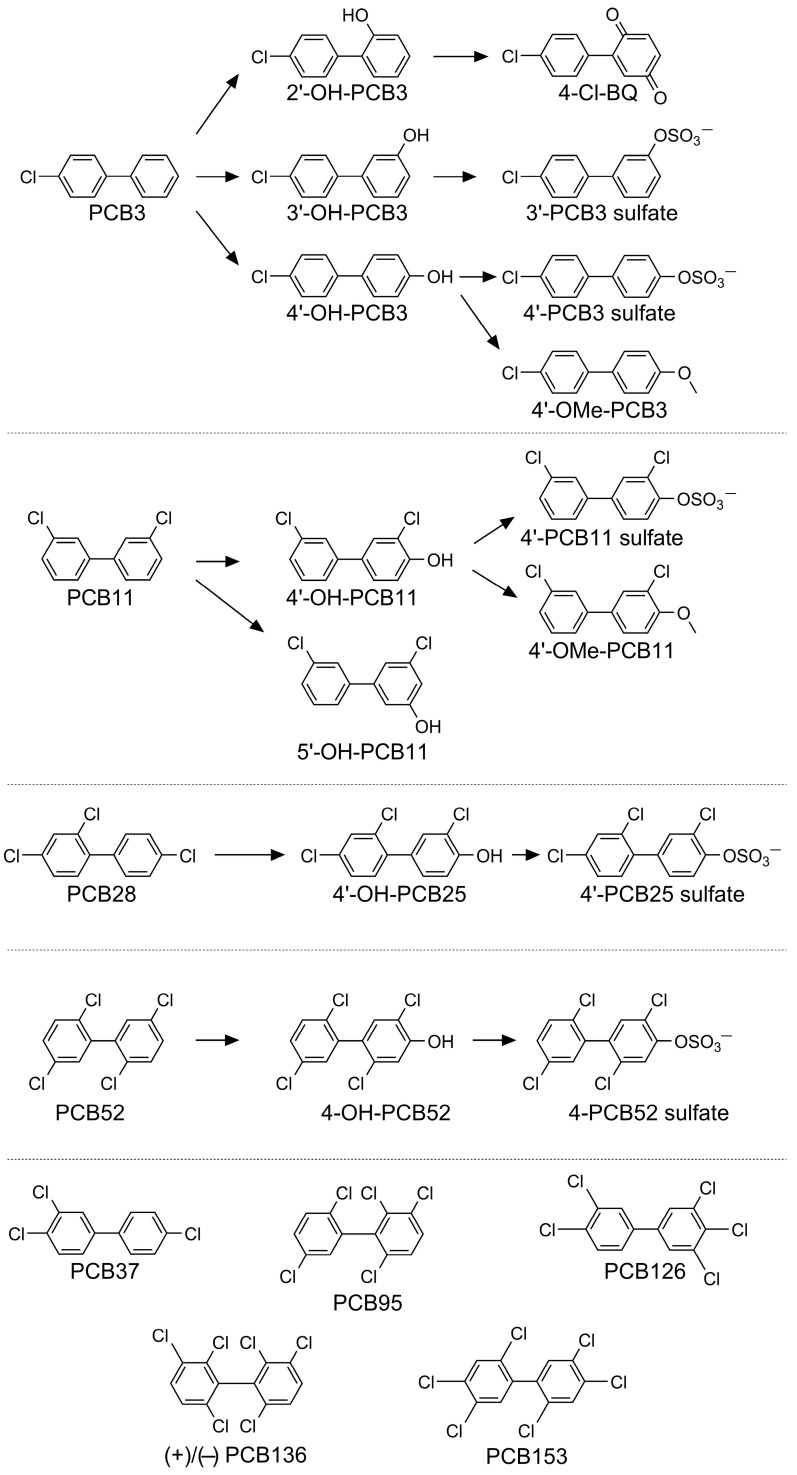
Chemical structure and abbreviation of the PCBs and their metabolites selected for morphological and behavioral testing in zebrafish. The selection included five lower-chlorinated PCBs (PCB3, PCB11, PCB28, PCB37, and PCB52) and their hydroxylated, methoxylated, and sulfated metabolites. The higher-chlorinated PCBs (≥5 chlorine atoms), including PCB95, PCB126, PCB153, and (+)- and (−)-PCB136 were also studied. Chemical names and abbreviations of PCBs and metabolites are listed in Table S1.

Details describing the synthesis of PCBs used in this study are described in the Supplementary Materials. All synthesis work was performed in the Synthesis Core of the Iowa Superfund Research Program (University of Iowa, Iowa City, IA, USA). The chemical structures of all test compounds and their corresponding abbreviations are shown in Figure 1. Their unique chemical identifiers are summarized in Table S1. All other chemicals were obtained from commercial sources and were of the highest purity available.

### 2.2. Zebrafish Husbandry

Adult wildtype tropical 5D fish and *cyp1a* reporter fish (Tg(*cyp1a*:nls-egfp)) [36] were used in this study. All zebrafish were maintained at the Sinnhuber Aquatic Research Laboratory (SARL), a dedicated aquatic research facility at Oregon State University, under approved Institutional Animal Care and Use Committee protocol 2024-0510. Fish were kept and group-spawned in densities of 400 fish/35-gallon tank (5D) and 16 fish/2.8 L tank (*cyp1a* reporter) at 28 °C with a 14:10 h light:dark cycle. Each spawning group contained a female:male population ratio of 2:1. Fish were maintained in a recirculating aquatic system supplemented with Instant Ocean salts (Spectrum Brands, Blacksburg, VA, USA) buffered to pH 7.4 with sodium bicarbonate. Routine health monitoring and feeding with Sparos Zebrafeed 300 µM occurred 2–3 times daily (Tecniplast, Buguggiate, Italy). Embryos were collected shortly after lights-on, between 8:00 and 9:00 a.m., and examined to select only fertilized, morphologically normal embryos at comparable developmental stages following established staging criteria [32]. At 4.5 h post-fertilization (hpf), embryos were selected and enzymatically dechorionated using pronase (Sigma Aldrich, St. Louis, MO, USA—#53702, CAS: 9036-06-0) [37]. At 6 hpf, embryos were individually transferred into 96-well, round-bottom, tissue culture–treated plates prefilled with 100 μL of embryo medium using an automated placement system. Embryos were kept at 28 °C in embryo medium containing 15 mM NaCl, 0.5 mM KCl, 1.3 mM CaCl_2_, 0.15 mM KH_2_PO_4_, 0.05 mM Na_2_HPO_4_, 2 mM MgSO_4_, and 4.7 mM NaHCO_3_ [38], buffered to pH 7.2.

### 2.3. Chemical Exposure

At 6–7 hpf, static chemical exposures were initiated. Twenty-eight compounds, including individual congeners, metabolites, and mixtures, were dispensed into individual wells, normalized to 1% DMSO, using an HP D300 Digital Dispenser (Hewlett Packard, Corvallis, OR, USA). Final nominal concentrations reflected the direct addition of each chemical to the 100 µL exposure volume in the wells. Embryos were exposed to 7 concentrations (0, 0.01, 0.1, 1, 10, 50, and 100 µM) with one row per concentration (*n* = 12) and a single chemical per plate. The selected concentration range was chosen to enable benchmark concentration modeling and to facilitate comparisons with previously published zebrafish developmental toxicity studies [39]. This exposure paradigm represents a standardized high-throughput screening approach used extensively to evaluate environmental chemicals. After chemical addition, all 96-well plates were sealed with ThermalSeal RTS (Excel Scientific, Victorville, CA, USA) to minimize evaporation and placed on an orbital shaker at 225–235 rpm for 18 h at 28 °C in the dark to ensure homogenous mixing. At 24 hpf, embryos were evaluated for embryonic photomotor response, then transferred to a static 28 °C incubator and kept in the dark for the remainder of the exposure period until 120 hpf, when larval photomotor response and morphology screening were conducted. Exposure solutions were not renewed during the experiment. Nominal concentrations are standard in high-throughput comparative screening formats and enable consistent, direct comparisons of relative toxicity across chemicals, as well as rapid hazard identification. Every plate carried a unique barcode, and all experimental metadata and endpoints were acquired and stored in the Zebrafish Acquisition and Analysis Program (ZAAP), a custom laboratory information management system [35].

### 2.4. Behavioral Assessments

#### 2.4.1. Embryonic Photomotor Response

At 24 hpf, embryos were tested for embryonic photomotor response (EPR). At this developmental stage, zebrafish embryos exhibit spontaneous contralateral tail contractions as spinal and tail musculature become innervated [32]. These movements are initiated by light stimuli perceived by photoreceptive cells located within the hindbrain [40]. The EPR assay was performed as previously described in [41]. This assay quantifies light-evoked tail contractions in zebrafish embryos as a measure of early sensorimotor and neurobehavioral function, where movement is quantified as changes in pixel intensity between sequential frames following light stimulation. Embryo movements were recorded continuously through the EPR assay, which consisted of four phases: (1) 30 s of background activity in darkness; (2) a 1 s pulse of visible light; (3) 9 s of darkness; (4) a second 1 s light pulse followed by 10 s of darkness. Videos were analyzed using a custom C# program that calculated a movement index for each frame based on pixel ratioing between consecutive frames. For downstream analysis, movement data were summarized over three defined intervals: 9 s prior to the first light pulse (background), 8 s following the first pulse (excitatory), and 7 s following the second pulse (refractory). Mean movement index values for each interval were used as behavioral response variables for statistical analysis. These intervals correspond to distinct neurobehavioral states, including baseline spontaneous motor activity (background), light-induced sensorimotor activation characterized by increased tail contractions (excitatory), and a reduced or absent response to repeated stimulation reflecting neural adaptation or refractory behavior (refractory). After the EPR assay was completed, 96-well plates were returned to 28 °C and kept in darkness for the remaining exposure duration.

#### 2.4.2. Larval Photomotor Response

At 120 hpf, larvae were assessed for locomotor activity using a larval photomotor response (LPR) assay conducted in ZebraBox behavior chambers (ViewPoint Life Sciences, Lyon, France). The assay evaluated putative neurobehavioral effects of chemical exposure by quantifying larval movement in response to alternating light–dark conditions. The LPR assay consisted of a 24 min protocol with four sequential cycles of 3 min of visible (VIS) light (1000 lux) followed by 3 min of infrared (IR) darkness. The initial cycle (6 min) served as an acclimation period and was excluded from analysis to minimize transient locomotor responses associated with handling and abrupt light transitions. Larval behavior from the remaining three cycles (18 min total) was used for downstream comparisons. Video tracking was performed using ZebraLab software (v5.33.0.440), which recorded movement every 40 ms. Total distance traveled was integrated into 6 s time bins over the analytical period. After data collection, wells containing dead or malformed larvae were excluded using a custom R script. Total movement for each larva was calculated, and treatment groups were compared with concurrent solvent controls.

### 2.5. Morphological Assessments

Following the EPR, the embryos are assessed for survival at 24 hpf, delays in developmental progression, altered spontaneous movement, and abnormal notochord development. Following completion of the LPR assay at 120 hpf, each larva was evaluated for a comprehensive suite of 10 developmental abnormalities. These included edema phenotypes such as yolk sac and pericardial edema; structural defects affecting the body axis, trunk length, caudal fin, and pectoral fin; abnormalities in pigmentation and somite formation; craniofacial and sensory malformations affecting the eyes, snout, and jaw; gross brain developmental defects; circulatory and notochord deformities; and assessments of swim bladder presence or inflation and touch responsiveness (see [42] for representative images of morphological anomalies). All endpoints were scored as binary (present or absent), consistent with established high-throughput zebrafish developmental toxicity screening approaches in which morphological abnormalities are recorded as discrete incidence-based outcomes to enable standardized scoring and statistical comparisons across large datasets [42]. To enable quantitative comparison of developmental toxicity across chemicals, individual morphological outcomes were consolidated into a summary endpoint termed “Any Effect,” defined as the occurrence of one or more developmental abnormalities in an individual larva. The lowest effect level (LEL) for each chemical was defined as the lowest concentration at which the percent affected was statistically significantly greater than the control group (binomial test, *p* < 0.05) [43]. Benchmark dose (BMD_10_) is the concentration that results in a 10% higher response (10% benchmark response) than the negative control. BMD_10_ values are provided in Supplementary Table S2.

### 2.6. cyp1a Reporter Line Expression

*cyp1a* induction was assessed using the Tg(*cyp1a*:nls-egfp) transgenic zebrafish line, which expresses enhanced green fluorescence protein (EGFP) via the *cyp1a* promoter [36]. This reporter line recapitulates *cyp1a* activation by AhR agonists with high sensitivity. PCB congeners and metabolites were selected by having a BMD_10_ value < 2 µM. For each of the 10 test compounds, 12 embryos were exposed to a single concentration selected to maximize the likelihood of observing AhR-mediated effects while maintaining sufficient survival for imaging analyses. Concentrations were chosen as the concentration immediately below the LEL identified in primary toxicity screens described above. Final exposure concentrations are listed in Supplementary Table S3. Chemical exposures were performed as described in Section 2.3. Briefly, compounds were dispensed into 96-well plates using an HP D300 Digital Dispenser, after which plates were sealed and shaken overnight under standard incubation conditions. Reporter embryos were exposed continuously, and *cyp1a* induction was evaluated at 120 hpf. This design is consistent with prior studies of the same reporter line using 8–12 embryos per compound at a single selected concentration for microscopy-based evaluation [44]. At each time point, six embryos per treatment were imaged using a Keyence BZ-X700 fluorescence microscope (Keyence USA, Itasca, IL, USA) equipped with a GFP filter. Imaging parameters were standardized across experiments (10× objective, 1/3 s exposure). EGFP expression was scored qualitatively by comparison with vehicle controls, and each embryo was assigned a binary value indicating the presence or absence of *cyp1a* induction.

### 2.7. Transcriptomic Sample Preparation and Data Processing

#### 2.7.1. Chemical Exposures for Transcriptomic Sampling

Embryos were subjected to the same dechorionation, handling, and 96-well plate preparation procedures described in Section 2.2. A subset of ten PCB congeners (PCB3, PCB11, PCB126), metabolites (2′-OH-PCB3, 3′-OH-PCB3, 4′-OH-PCB3, 4-OH-PCB11, 5-OH-PCB11), and mixtures (FRM, HR-PCB) was selected for transcriptomic analysis based on their developmental toxicity profiles and representative coverage of major mechanistic classes identified in preliminary screening. All chemical stocks were prepared in 100% DMSO, enabling automated dispensing with an HP D300 Digital Dispenser [45]. Compounds were dispensed into individual wells at 6 hpf into 100 μL of embryo medium, with all wells normalized to 1% DMSO. For each compound, a single exposure concentration was selected to achieve approximately 80% incidence of cumulative morphological effects (EC_80_) at 120 hpf, informed by BMD_10_ data. Embryos were statically exposed from 6 to 48 hpf, a timeframe chosen to align with published studies and to capture transcriptional changes preceding the onset of overt morphological effects [46]. Following chemical dispensing, plates were sealed, placed on an orbital shaker (235 rpm), and maintained in darkness at 28 °C for the duration of the exposure. For each chemical and its matched control, only phenotypically normal embryos were collected into four pools of eight embryos (*n* = 4). To ensure phenotypic anchoring, holdback plates (*n* = 16 per chemical) were maintained and assessed for morphological effects at 120 hpf. This early sampling window was selected to capture transcriptional responses during the period of highest developmental susceptibility, prior to the emergence of overt morphological abnormalities.

#### 2.7.2. RNA Collection and Isolation

Pooled embryos were transferred to 1.5 mL twist-cap microtubes (TUBE1R5-S; Next Advance, Troy, NY, USA) and euthanized on ice. After removing excess water, 200 μL of 1× RNAshield (Zymo Research, Irving, CA, USA) was added following 15 min on ice. Samples remained at room temperature for up to 15 min before storage at −20 °C. Four pools of eight embryos were collected per treatment group and corresponding controls. For RNA isolation, tubes were thawed at 32 °C for 30 min, followed by addition of 100 μL of 0.5 mm zirconium silicate beads (BioSpec, Bartlesville, OK, USA). Homogenization was performed using a Bullet Blender Storm Pro (Next Advance, Troy, NY, USA) at speed 10 for 5 min. Samples were centrifuged at 12,000× *g* for 5 min, and 250 μL of supernatant was aliquoted into two new plates (totaling 500 μL). RNA was extracted using the KingFisher Apex (Thermo Fisher Scientific, Waltham, MA, USA) automated system with the Zymo Quick RNA MagBead Kit and eluted in 50 μL RNase/DNase-free water. RNA integrity was assessed using an Agilent Tapestation 4200 (Agilent Technologies, Santa Clara, CA, USA), while RNA quantity was measured using ThermoFisher QuantIT (Thermo Fisher Scientific, Waltham, MA, USA); only samples with RIN > 8 were used for sequencing. In total, 48 samples, including 8 control replicates, were prepared for RNA-seq.

#### 2.7.3. RNA Sequencing and Alignment

mRNA sequencing was performed on four biological replicates per exposure. Library preparation and sequencing were conducted by Lexogen (Greenland, NH, USA) using the QuantSeq Pool Sample Barcoded 3′ mRNA-Seq workflow, with 120 ng RNA input per sample and pools of 12 samples. Libraries were sequenced to a target depth of approximately 15 million reads per sample. All raw and processed data were deposited in the NCBI Gene Expression Omnibus (GEO), accession GSE326509. Quality assessment of fastq files was performed using FastQC [47], and reads were trimmed with Trimmomatic [48] to remove low-quality bases (Phred < 25) and adaptor sequences. Trimmed reads were aligned to the *Danio rerio* v11 genome (RefSeq GCF_000002035.6) using STAR [49] with the following parameters: --outFilterType BySJout --outFilterMultimapNmax 20 --readFilesCommand zcat --alignSJoverhangMin 8 --alignSJDBoverhangMin 1 --outFilterMismatchNmax 999 --outFilterMismatchNoverLmax 0.6 --alignIntronMin 20 --alignIntronMax 1,000,000 --alignMatesGapMax 1,000,000 --outSAMattributes NH HI NM MD --outSAMtype SAM. Resulting SAM files were converted to gene-level count files using HTSeq [50] with parameters: -f sam -a 10 -t gene -i gene_id. RNA-seq data quality was assessed using multiple quality control metrics. Total sequencing depth per sample ranged from 16 to 24 million reads, with uniquely mapped read percentages consistently high (~90%). Following UMI-based collapsing, 5–7 million reads per sample were retained.

#### 2.7.4. Differential Expression Analysis

Raw count files were normalized using DESeq2 [51], which was also used for differential gene expression analysis. Gene counts from all 8 control samples were concatenated to create a single global control dataset, used as the normalization reference for all PCB samples. Differentially expressed genes (DEGs) were defined using a *p*-value ≤ 0.05, without applying a fold-change cutoff, to allow for broad, exploratory identification of gene expression changes. Principal component analysis (PCA) was conducted using the DESeq2 plotPCA function with variance-stabilized data. Overlap among DEG lists was assessed using InteractiVenn [52]. Heatmaps were generated using Rstudio (version 2026.01.2; Build 418) (heatmap.2, aheatmap, ggplot) with Euclidean distance and ward.D2 clustering. Functional enrichment was performed using g:Profiler [53], querying GO terms, KEGG, Reactome, regulatory motifs, protein databases, and human phenotype ontology.

### 2.8. ToxPi Construction and Data Integration

Physicochemical descriptors for all test compounds were calculated using ChemSketch (v2025; ACD/Labs, Toronto, ON, Canada). The following descriptors were included: molecular weight, logP, logS, density, polarizability, molar volume, surface tension, and percent elemental composition (C, H, Cl, O, and S). Structural descriptors, including the number of ortho-, meta-, and para-chlorines, total chlorine count, and ortho-, meta-, and para-substituted non-chlorine alkyl groups (e.g., hydroxyl, sulfooxy), were manually counted from chemical structures. These physicochemical parameters were incorporated into the ToxPi model as slice-level quantitative attributes. Biological data streams incorporated into the ToxPi model included morphological toxicity, EPR, LPR, and RNA-seq (differentially expressed genes at 48 hpf). All biological and physicochemical inputs were scaled across the chemical set to ensure comparability, and, where necessary, values were inverted so that higher values uniformly indicated greater biological or physicochemical signal strength.

Integrated scoring and visualization were performed using ToxPi 2.0 [29]. Each slice of the ToxPi profile represented one data domain (physicochemical, morphology, EPR, LPR, RNA-seq), and all slices were assigned equal weight. Slice size was proportional to each chemical’s scaled value for that domain, allowing direct comparison of multi-endpoint profiles across chemicals. ToxPi scores were then used to rank chemicals, examine relative biological activity, and evaluate relationships between chemical characteristics and effects across behavioral, morphological, and transcriptomic domains. KEGG pathway enrichment results were organized using the KEGG BRITE hierarchy, which groups individual pathways into nested, higher-order functional categories (e.g., metabolism, signal transduction, organismal systems). This approach reduces redundancy among overlapping pathways and facilitates systems-level interpretation of pathway alterations. Hierarchical clustering of ToxPi profiles was performed using Ward’s method within the ToxPi GUI to identify chemicals with similar multi-endpoint signatures. This integrated ToxPi approach enabled simultaneous evaluation of physicochemical properties and early-life biological effects, providing a unified framework for identifying clusters of PCBs with similar mechanistic signatures and contrasting chemicals across multiple biological and structural domains.

### 2.9. Statistical Analyses

For morphological data, binary incidence data (0/1) recorded per well by ZAAP were analyzed using custom R code, as described [43]. Statistical significance thresholds were estimated by Fisher’s exact test with a family-wise error rate. The EPR activity within the Background (B), Excitatory (E), and Refractory (R) intervals was compared to the negative control using a percent change threshold (≥50% peak difference) and a Kolmogorov–Smirnov test (Bonferroni-corrected *p* < 0.007; 0.05 ÷ 7 concentrations). For LPR, the area under the curve was computed per plate and averaged per chemical. Only the 3rd light/dark cycle was analyzed, comparing treatment responses to the control by two-sample K-S test (*p* < 0.01); a concentration was considered active if *p* < 0.01 and the AUC treatment:control ratio was ≥1.0 or ≤−0.3. Animals dead or malformed at 120 hpf were excluded. All statistical analyses were carried out in RStudio (version 2026.01.1).

## 3. Results and Discussion

### 3.1. Morphology and Behavior

**Figure 2:**
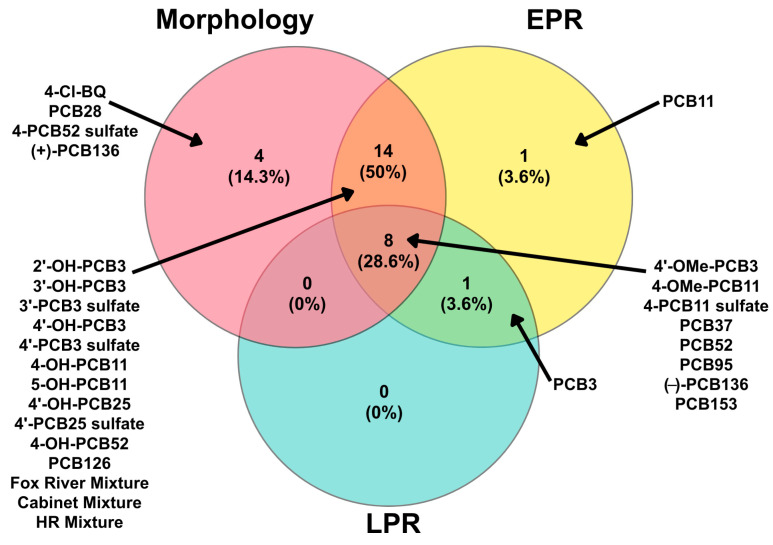
Venn diagram for the number of chemicals that had an effect on morphology, embryonic photomotor response (EPR), and larval photomotor response (LPR). Morphology effect indicates a chemical had a BMD_10_ reported for the chemical in “Any Effect”, EPR effect indicates a chemical had a BMD_10_ reported in EPR, and LPR effect indicates a chemical had a BMD_10_ reported in LPR. Numbers in parentheses represent the percentage of tested compounds that fall within a given region.

The developmental toxicity and neurotoxicity of a suite of parent PCBs, hydroxylated, methoxylated, and sulfated PCB metabolites, as well as environmentally relevant mixtures (Figure 1), were evaluated in embryonic zebrafish using morphological and behavioral endpoints. Across all PCBs tested, effects were observed in morphology, EPR, and LPR. BMD_10_ modeling was used to quantify potency across endpoints, revealing variability in sensitivity among PCBs. The Venn diagram in Figure 2 summarizes the overlap among these endpoints. Morphological effects were the most frequently observed outcome, with 26 of the 28 PCBs producing at least one morphological abnormality. Four PCBs caused morphology-only effects without measurable behavioral disruption, while only PCB11 exhibited effects exclusively in EPR. No PCBs produced effects solely in LPR. Fourteen of the PCBs induced both morphological and EPR effects, and eight PCBs produced effects across all three domains, indicating shared developmental and neurobehavioral toxicity. No overlap was observed between morphology and LPR in the absence of EPR effects, suggesting that larval behavioral alterations generally co-occurred with earlier photomotor or morphological effects.

**Figure 3:**
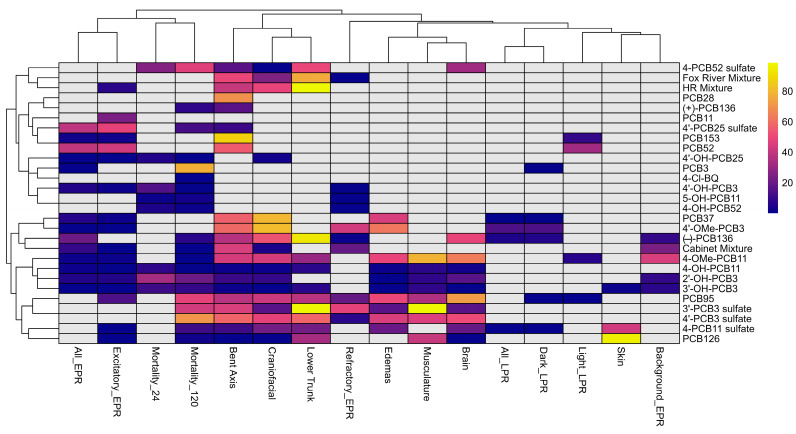
Heatmap of all endpoints across both models for each individual chemical and mixture. Colored boxes represent the BMD_10_ for each endpoint. BMD_10_ values for each endpoint can be found in Table S3.

A heatmap of individual endpoints is shown in Figure 3, revealing two clusters of PCB mixtures, individual PCB congeners, and PCB metabolites across all developmental endpoints. Across both clusters, PCB congeners with varying degrees of chlorination and numbers of ortho substituents exhibited similar response profiles. Notably, compounds spanning a range of chlorination degrees and substitution patterns were distributed within the same clusters rather than being separated by these structural features. Because the degree of chlorination and the substitution pattern are frequently used to predict nuclear transcription factor-mediated effects implicated in PCB toxicity [54], this clustering pattern indicates that these features alone do not explain the observed variation in morphological and behavioral outcomes in zebrafish in vivo.

One cluster included most hydroxylated, methoxylated, and sulfated PCB3 and PCB11 metabolites, except for 5-OH-PCB11 and 4′-OH-PCB3. This cluster also included several parent PCBs with known neurotoxic potential, including PCB37 [55], PCB95 [21,56,57], PCB126 [58,59], and (−)-PCB136 [58,59], as well as the Cabinet Mixture [60]. These PCB compounds exhibited a broad range of effects across morphological and behavioral endpoints. These compounds had low BMD_10_ values for the bent axis, craniofacial, and lower-trunk defects, as well as edema, musculature, and brain abnormalities. Additionally, these compounds also showed effects on EPR and LPR measurements.

The second cluster in the hierarchical clustering analysis included the higher-chlorinated hydroxylate and sulfated PCB metabolites, PCB3, PCB11, PCB28, PCB52, (+)-PCB136, and PCB153; the FRM and HR-PCB mixtures; and three lower-chlorinated PCB metabolites, 4′-OH-PCB3, 4-Cl-BQ, and 5-OH-PCB11. Interestingly, 4-Cl-BQ, a highly reactive and toxic PCB metabolite [18], affected mortality at 120 hpf but altered no other endpoints investigated. The PCB compounds in this cluster affected EPR outcomes, mortality, and, for 4-PCB52 sulfate, the FRM and HR-PCB mixtures, bent-axis, craniofacial, and lower-trunk defects. Compounds in this cluster typically had no effect on other morphological and LPR outcomes. Importantly, this cluster included both lower- and higher-chlorinated congeners and metabolites, further supporting that clustering was not determined solely by degree of chlorination or substitution pattern.

Several PCB congeners included in this study have previously been associated with developmental and neurodevelopmental toxicity across multiple experimental models. Zebrafish exposed to PCB126 exhibited craniofacial malformations [61]. Developmental exposure to PCBs can alter dendritic and axonal patterning, contributing to neurodevelopmental abnormalities [62]. Effects on neuronal morphology have been observed for PCB11, 4-OH-PCB11, and 4-PCB11 sulfate, with increased axonal and dendritic growth in cortical and hippocampal neurons and enhanced dendritic arborization in developing rat neurons [63,64,65]. This also aligns with altered dendritic and axonal morphology and apoptosis in primary rat cortical cultures exposed to PCB37 [55]. In rodent models, PCB95 enhances dendritic growth and arborization in hippocampal neurons through ryanodine receptor–dependent signaling and altered calcium dynamics [21,56,57].

Beyond cellular effects, developmental PCB exposure has also been linked to functional neurobehavioral outcomes. For example, Zebrafish developmentally exposed to PCB95 exhibited altered locomotor behavior in the LPR, showing dose- and time-dependent effects [66]. Developmental exposure to PCB126 impaired behavioral habituation and produced developmental abnormalities such as edema and craniofacial defects [67,68,69], while PCB153 resulted in delayed startle responses in zebrafish [70]. In rat adrenal cultures, PCB153 exposure disrupted dopaminergic signaling, suggesting interference with neurotransmitter regulation [71]. Adult mice exposed to the HR-PCB mixture exhibited deficits in long-term spatial memory [72]. Mice developmentally exposed to the FRM altered social communication and social novelty [73].

One intriguing finding is that the (−)-PCB136 atropisomer clustered most closely with the Cabinet Mixture, while the (+)-PCB136 atropisomer clustered with PCB28, suggesting atropisomer-dependent differences in biological activity. The observation that (−)-PCB136 produced responses across several endpoints, whereas (+)-PCB136 produced minimal effects, is consistent with previous in vitro studies demonstrating enantioselective toxicity. Specifically, (−)-PCB136 sensitizes ryanodine receptors and promotes dendritic growth in neuronal cultures, while the (+)-enantiomer shows little or no activity [58,59]. Other chiral PCB congeners, such as PCB95, also atropselectively affect outcomes relevant to PCB neurotoxicity in in vitro and cell culture models [74]. Thus, this study, for the first time, demonstrated the enantioselective effects of PCB136 atropisomers in vivo on outcomes relevant to developmental neurotoxicity.

Together, these results demonstrate that PCB metabolites, particularly hydroxylated and sulfated metabolites, adversely affect developmental and neurobehavioral outcomes more frequently and at lower concentrations than the parent PCB congeners. The clustering patterns further indicate that structural features, stereochemistry, and metabolic transformation influence toxicity profiles. The overlap between morphological and behavioral responses highlights the value of integrated phenotypic screening approaches that capture both structural and functional endpoints when assessing the developmental toxicity of PCBs.

### 3.2. cyp1a Reporter Expression

PCBs can cause toxic effects through activation of the aryl hydrocarbon receptor (AhR), which results in the induction of *cyp1a* in vertebrate animals. Several PCB metabolites have also been shown to induce AhR activity in rat cells in culture [13,75,76]. Therefore, the spatial induction of *cyp1a* was evaluated using a *cyp1a* reporter zebrafish line for a targeted subset of chemicals selected based on potency in the developmental toxicity screen. Nine PCBs (PCB126 and (-)-PCB136), and PCB metabolites (3′-OH-PCB3, 4′-OH-PCB3, 4-Cl-BQ, 4-OH-PCB11, 5-OH-PCB11, 4′-OH-PCB25, and 4-OH-PCB52) with BMD_10_ values below 2 µM, along with 4′-PCB25 sulfate, were selected for follow-up assessment of *cyp1a* activation. Embryos were exposed beginning at 6 hpf and imaged at 120 hpf under exposure and imaging conditions optimized to detect robust *cyp1a* induction.

**Figure 4:**
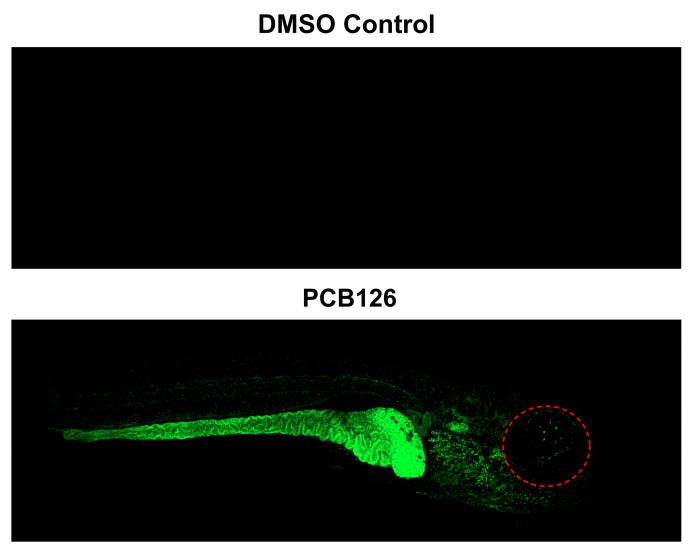
Lateral fluorescence images of a *cyp1a* reporter line (Tg(*cyp1a*:nls-egfp)) at 120 hpf following embryonic exposure to 0.099 µM PCB126, imaged from right. The red dashed circle indicates the eye of the zebrafish. Max exposure = 3000.

At these relatively high exposure concentrations, only PCB126 activates the AhR and elicits strong *cyp1a* activation above control levels (Figure 4). PCB126 exposure resulted in strong GFP expression localized primarily to the intestinal and liver region, consistent with canonical AhR-mediated responses [77]. None of the remaining parent PCBs or metabolites induced *cyp1a* expression distinguishable from controls at the exposure concentrations tested, despite exhibiting morphological and/or behavioral effects in the screening assays (Figure 3). These results indicate that, within this subset of potent developmental toxicants, *cyp1a* induction was restricted to PCB126, suggesting that AhR activation may not be the primary mechanism underlying the majority of observed PCB-associated developmental effects. Although only PCB126 induced *cyp1a* expression in this study, future investigations incorporating full concentration–response analyses may be necessary to characterize AhR activation thresholds and determine the contribution of AhR signaling to PCB-induced developmental toxicity.

### 3.3. Transcriptomics

#### 3.3.1. Overall Transcriptomic Response

**Figure 5:**
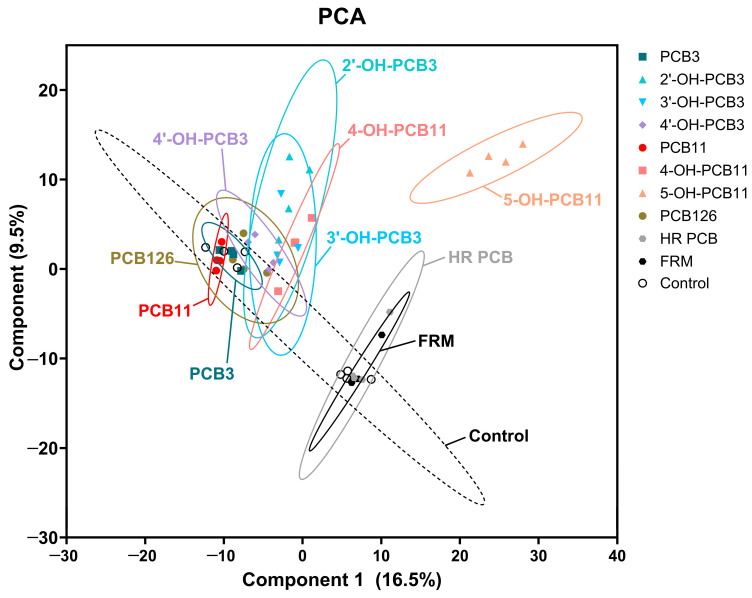
Principal component analysis (PCA) plot of gene expression profiles across exposure groups. Each point represents an individual sample, colored and shaped by exposure group. Ellipses denote 95% confidence intervals, illustrating exposure-specific transcriptional responses and overlap among related PCB exposures.

Global transcriptional patterns for 10 compounds across PCB, hydroxylated PCB, and mixture exposures were evaluated using principal component analysis (PCA) of normalized read counts (Figure 5). Samples clustered according to exposure, with parent congeners and most metabolites exhibiting profiles similar to controls. One metabolite, 5-OH-PCB11, produced a unique response from controls and all other tested compounds, while 2 mixtures, HR-PCB and FRM, produced a unique response from PCBs and metabolites. Differential expression analysis revealed a wide range of responses across exposures. Parent PCB congeners elicited few differentially expressed genes (DEGs), including PCB3 (4 DEGs), PCB11 (8 DEGs), and PCB126 (10 DEGs). Among hydroxylated PCB3 metabolites, 2′-OH-PCB3 produced the largest transcriptional response with 165 DEGs, followed by 3′-OH-PCB3 with 62 DEGs. In contrast, 4′-OH-PCB3 induced only 13 DEGs, highlighting the importance of hydroxylation position in modulating gene expression responses.

Hydroxylated PCB11 metabolites exhibited diverse effects. Exposure to 4-OH-PCB11 resulted in 56 DEGs. 5-OH-PCB11 elicited a strong transcriptional response, with 958 DEGs, including 433 upregulated and 525 downregulated genes, consistent with its distinct separation from controls in the PCA. Mixture exposures also had moderate effects on transcriptomics. The HR-PCB mixture and FRM both induced 51 DEGs. Among all 10 of the PCBs tested for transcriptomic effects, a total of 1078 unique DEGs were identified, with 582 being exclusively downregulated and 496 exclusively upregulated.

#### 3.3.2. Upregulated Genes Following PCB Exposure

**Figure 6:**
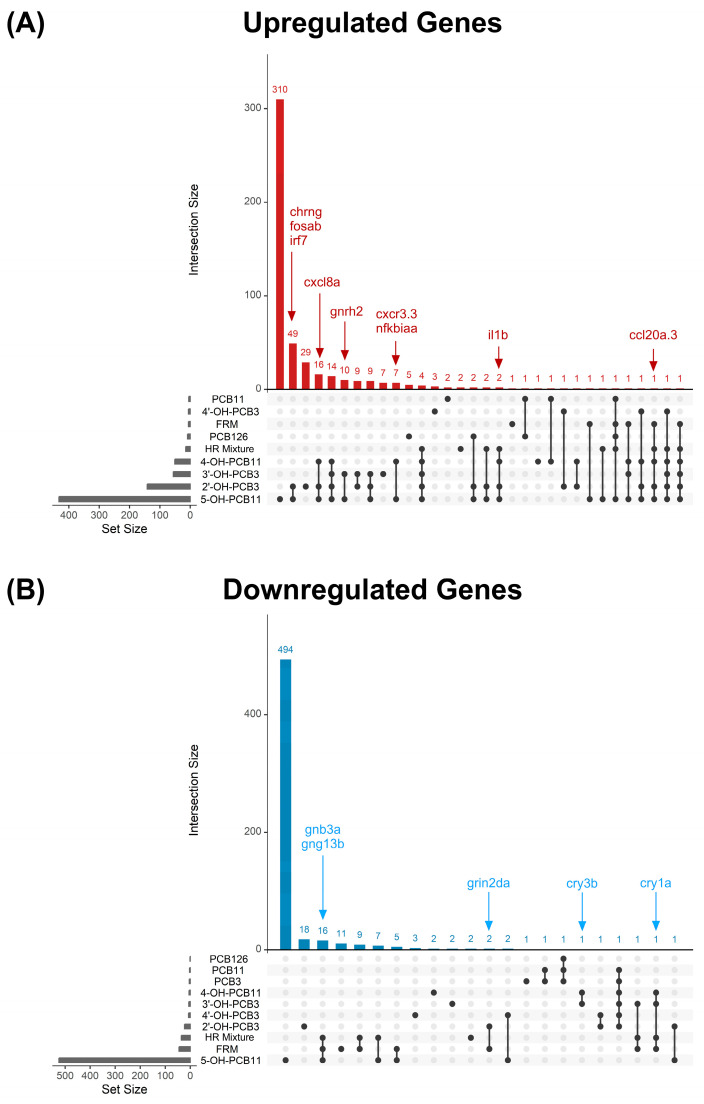
(**A**) Upset plot showing distinct and overlapping genes upregulated by PCB exposure in zebrafish larvae. (**B**) Upset plot showing distinct and overlapping genes downregulated by PCB exposure in zebrafish larvae.

To characterize PCB-dependent transcriptional responses in zebrafish, an UpSet plot was used to compare upregulated genes across individual PCB congeners, hydroxylated PCB metabolites, and PCB mixtures, identifying shared and exposure-specific molecular signatures (Figure 6A). Across exposures, there was a robust induction of genes involved in immune responses. The chemokine *ccl20a.3* expression was upregulated following exposure to the FRM and HR mixtures as well as 4-OH-PCB11, 2′-OH-PCB3, and 5-OH-PCB11, consistent with human epidemiological data demonstrating a positive correlation between *ccl20* expression and both non–dioxin-like and dioxin-like PCB exposure [78]. Similarly, *cxcl8a,* also known as IL-8, expression was upregulated by 4-OH-PCB11, 2′-OH-PCB3, and 5-OH-PCB11, aligning with evidence of a positive association between PCB body burden and IL-8 levels in humans [79]. The pro-inflammatory cytokine *il1b* expression was also induced by the HR-PCB mixture, 4-OH-PCB11, 2′-OH-PCB3, and 5-OH-PCB11, consistent with previous findings showing increased IL-1β in mice exposed to low-chlorinated PCBs [80] and in human cells exposed to Aroclor 1245 [81]. Additional immune-related genes, including *cxcr3.3*, *irf7*, and *nfkbiaa*, were also upregulated; however, direct evidence linking these transcriptional responses to PCB exposure remains limited, suggesting potential yet underexplored roles for these pathways in PCB-induced immune modulation.

In addition to immune-related pathways, several genes associated with neuroactive signaling and neuronal activity were also differentially expressed following PCB-related exposures. The nicotinic acetylcholine receptor subunit *chrng* and the neuropeptide hormone *gnrh2* were upregulated following exposure to hydroxylated lower-chlorinated PCB metabolites. Direct evidence linking PCB exposure specifically to regulation of *chrng* is limited; however, PCBs are known to disrupt cholinergic signaling more broadly [82,83], indicating this gene may represent a previously underrecognized target of PCB metabolites. A study examining *gnrh* observed a significant increase following Aroclor 1221 exposure in mouse hypothalamic cells [84]. Additionally, *fosab*, a member of the fos immediate-early gene family [85], was upregulated following exposure to 2′-OH-PCB3 and 5-OH-PCB11. These immediate-early transcription factors are markers of neuronal activation and are implicated in long-term plasticity and gene expression programs following synaptic input [86,87]. Although direct evidence of *fos* regulation following exposure to PCBs and their metabolites remains limited, PCBs have been shown to broadly alter neural gene expression and disrupt pathways associated with synaptic function and neurotoxicity in mouse models [88,89]. These suggest that PCB metabolites may similarly influence neuronal signaling and activity-dependent transcriptional responses.

#### 3.3.3. Downregulated Genes Following PCB Exposure

An UpSet plot examining the overlap in downregulated genes is shown in Figure 6B. Across PCBs and hydroxylated PCBs, several genes involved in neuroactive signaling and neuronal activity were downregulated. Both *gnb3a* and *gng13b*, which encode β and γ subunits of heterotrimeric G proteins that mediate G protein-coupled receptor (GPCR) signaling [90], were downregulated following exposure to the HR mixture, FRM, and 5-OH-PCB11. These findings are consistent with earlier results demonstrating that PCBs alter neurotransmitter systems that rely on GPCR signaling, including dopaminergic and adrenergic pathways [91,92,93], suggesting that reduced expression of these genes may reflect broader disruption of neuronal signal transduction. Additionally, *grin2da*, which encodes a subunit of the NMDA-type glutamate receptor involved in excitatory neurotransmission and synaptic plasticity [94], was downregulated following exposure to 2′-OH-PCB3 and FRM. While evidence of *grin2da* is limited following PCB exposure, PCBs alter glutamatergic signaling and NMDA receptor–dependent calcium pathways that regulate neuronal development and synaptic plasticity [10,56], supporting a potential role for PCB metabolites and mixtures in altering excitatory neurotransmission.

Notably, multiple core circadian clock components, including *cry1a* and *cry3b* [95] were downregulated across several PCB exposure conditions. Alteration of circadian regulators has been reported following PCB exposure in zebrafish and mammalian systems [91,92,93] and is thought to reflect disruption of endocrine and metabolic signaling pathways that interface with the molecular clock [96,97]. While the analysis did not include time-resolved or functional assays of circadian rhythmicity, the downregulation of clock-associated genes across exposures suggests that PCB exposure may influence molecular components of the circadian system. Circadian gene downregulation has been associated with altered metabolic homeostasis, stress responses, and behavioral outcomes, suggesting that PCB-induced suppression of clock genes may represent a molecular event linking PCB exposure to downstream physiological dysfunction.

#### 3.3.4. Pathway Analysis Following PCB Exposure

**Figure 7:**
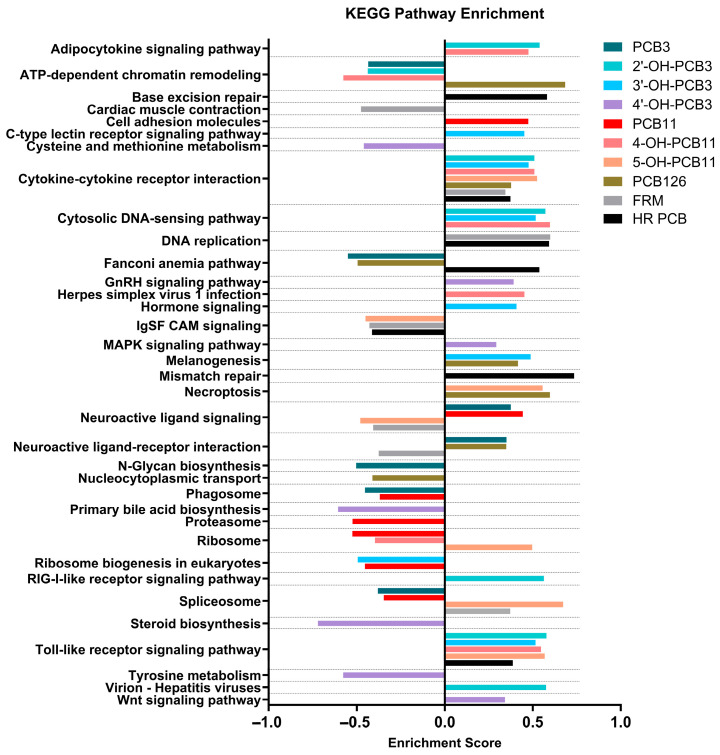
The top seven Kyoto Encyclopedia of Genes and Genomes (KEGG) pathways significantly enriched following exposure to individual PCB congeners, PCB metabolites, and PCB mixtures in zebrafish. Pathway enrichment analysis was performed separately for each exposure condition, and the seven most significantly enriched pathways are shown for each group. Enriched pathways reflect biological processes and signaling networks altered by PCB-related exposures, highlighting both shared and exposure-specific metabolic and molecular responses in zebrafish.

KEGG pathway enrichment analysis across PCB congeners, metabolites, and mixtures revealed similarities and differences among tested compounds (Figure 7). Across exposure conditions, there was consistent enrichment of innate immune and inflammatory signaling pathways, most prominently cytokine–cytokine receptor interaction and Toll-like receptor pathways. Activation of these pathways suggests a conserved immune-stress response [98,99] to PCB exposure in zebrafish in vivo. As discussed above, upregulated genes, including *ccl20a.3*, *cxcl8a*, *il1b, cxcr3.3*, *irf7*, and *nfkbiaa*, contributed to pathway alterations. Studies have previously reported that PCBs induce the cytokine-cytokine receptor pathway in human preadipocytes [100,101]. In contrast, enrichment of neuroactive ligand signaling and neuroactive ligand–receptor interaction pathways exhibited bidirectional regulation in the present study, with pathway activation observed for some PCB congeners (e.g., PCB3 and PCB11) and repression observed for others, including the hydroxylated metabolite 5-OH-PCB11 and the FRM mixture. As discussed above, genes involved in these pathway alterations include upregulation of *chrng*, *gnrh2*, and *fosab* and downregulation of *gnb3a*, *gng13b*, *grin2da*. These findings indicate that PCB-associated disruption of neuroactive ligand signaling is not uniform across congeners or metabolites. Similarly, bidirectional effects on neuroactive ligand signaling, including glutaminergic [102] and dopaminergic [103,104], have been reported in the PCB literature and may be attributed to differences in congener structure, receptor affinity, or downstream signaling cascades, particularly during neurodevelopment. Furthermore, PCBs are known endocrine disruptors [105,106], with pathways such as gonadotropin-releasing hormone (GnRH) signaling, primary bile acid biosynthesis, and steroid biosynthesis altered by 4′-OH-PCB3 in this study. The GnRH signaling pathway has been altered following PCB126 exposure in zebrafish [107]. While evidence of bile acid pathway alterations in zebrafish following PCB exposure is limited, mice exposed to the FRM mixture exhibited alterations in primary bile acids following exposure [108]. Zebrafish embryos exposed to PCB77 exhibited altered steroid hormone biosynthesis pathways [109].

### 3.4. ToxPi Analysis

**Figure 8:**
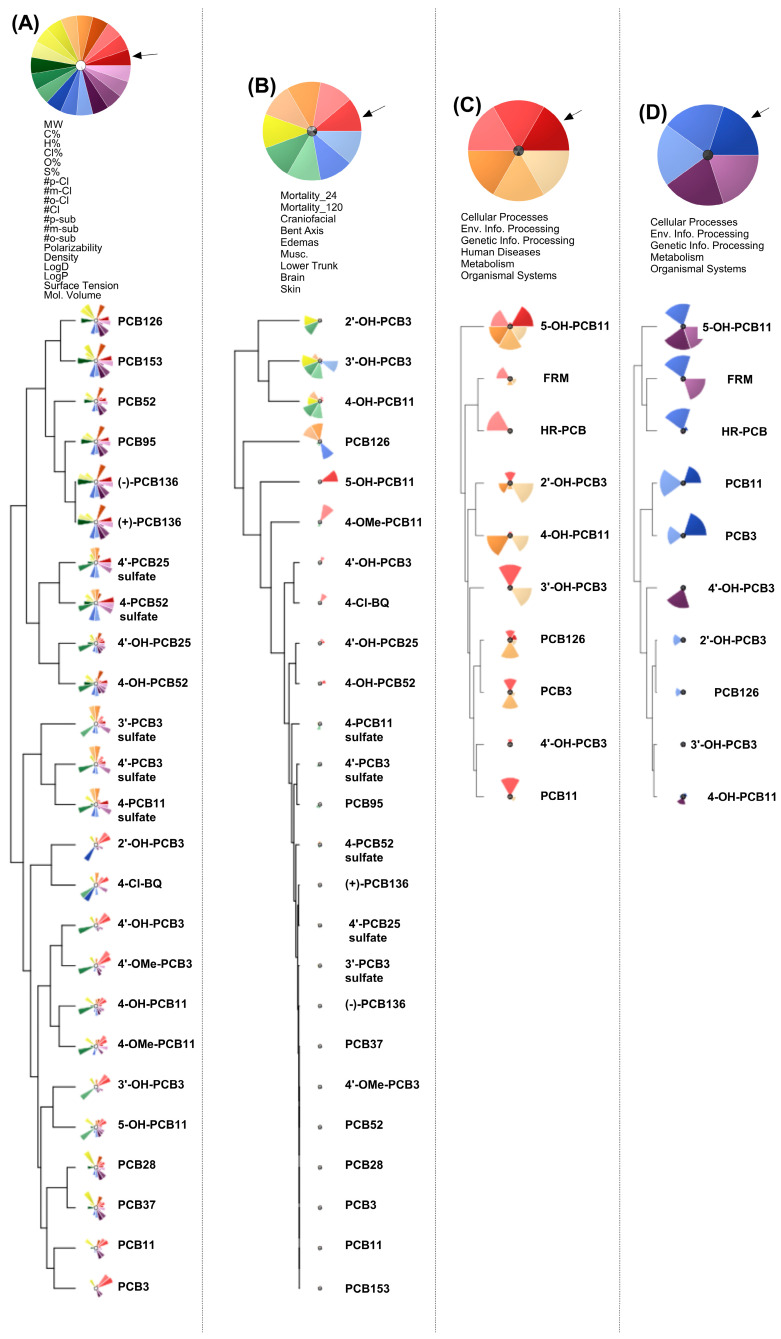
Multidimensional clustering of PCB congeners (PCB3, PCB11, PCB126), metabolites (2′-OH-PCB3, 3′-OH-PCB3, 4′-OH-PCB3, 4-OH-PCB11, 5-OH-PCB11), and mixtures (FRM, HR-PCB) using integrated physicochemical, toxicity/morphology, and pathway-level responses. Hierarchical clustering was performed using Toxicological Prioritization Index (ToxPi) profiles generated for each compound evaluated in this study, with each ToxPi corresponding to one parent PCB, metabolite, or mixture. (**A**) Physicochemical properties. ToxPi slices represent scaled physicochemical descriptors, including molecular weight, elemental composition, degree and position of chlorination and substitution, polarizability, density, LogD, LogP, surface tension, and molecular volume. (**B**) Mortality and morphology. ToxPi slices summarize in vivo toxicity and morphological outcomes, including 24 h and 120 h mortality, craniofacial defects, bent axis, edema, muscle defects, and effects on the lower trunk, brain, and skin. (**C**) Activated pathways. Clustering based on enriched biological pathways mapped to the KEGG hierarchy, grouped into major functional classes (cellular processes, environmental information processing, genetic information processing, human diseases, metabolism, and organismal systems). (**D**) Suppressed pathways. Clustering based on downregulated KEGG pathways using the same hierarchical classification scheme as in (**C**). For all ToxPi diagrams, individual slices correspond to specific data domains within each analysis, and the slice area is proportional to the relative magnitude of the response for a given compound. The arrows indicate the ordering of slices, proceeding counter-clockwise. Pathway-based clustering in panels (**C**,**D**) follows KEGG hierarchical organization.

Hierarchical clustering based on physicochemical descriptors (Figure 8A) revealed structure–property relationships that separated parent PCBs from their hydroxylated, methoxylated, and sulfated metabolites. Parent congeners clustered primarily by molecular weight, degree of chlorination, hydrophobicity (LogP/LogD), and polarizability, with higher-chlorinated non-dioxin-like PCBs (PCB52, PCB95, PCB136 atropisomers, and PCB153) clustering together with the dioxin-like congener PCB126, whereas lower-chlorinated PCBs and PCB metabolites formed a separate cluster. Both PCB136 atropisomers clustered closely with structurally similar non-coplanar congeners. In contrast, hydroxylated, methoxylated, and sulfated metabolites formed distinct subclusters driven by increased polarity, altered surface tension, and reduced hydrophobicity. Sulfated metabolites did not cluster independently of their parent structures; instead, 4′-PCB25 sulfate and 4-PCB52 sulfate clustered closely with their corresponding hydroxylated metabolites (4′-OH-PCB25 and 4-OH-PCB52), while sulfates derived from lower-chlorinated PCBs formed a separate cluster. These results demonstrate that relatively small structural modifications (e.g., hydroxylation or sulfation) substantially affect clustering by physicochemical properties, supporting the idea that metabolism alters PCB bioactivity potential beyond the parent-compound structure alone.

Clustering based on morphological endpoints (Figure 8B) produced groupings that only partially overlapped with physicochemical clusters, indicating that structural similarity alone is insufficient to predict organism-level toxicity. Notably, several parent congeners with similar physicochemical properties displayed divergent morphological signatures. For example, PCB126 and PCB153 were separated based on craniofacial, axis, and brain defects. Several PCB metabolites clustered together across multiple domains, including edema and muscle defects, for a subset of hydroxylated lower-chlorinated PCBs (2′-OH-PCB3, 3′-OH-PCB3, 4-OH-PCB11). Conversely, sulfated metabolites, despite clustering tightly in physicochemical space, exhibited relatively stronger morphological perturbations and clustered distally from parent PCB congeners. These findings highlight differences between physicochemical similarity and functional toxicity, emphasizing that morphology-based clustering captures biological effects that are not readily inferred from chemical descriptors alone.

Clustering based on activated pathways from the 10 compounds that underwent transcriptomic analysis using the BRITE hierarchy (Figure 8C) revealed convergence of structurally diverse compounds on shared biological processes. Among the 10 tested compounds, 5-OH-PCB11 clustered separately from all other PCB compounds, consistent with the PCA (Figure 5). Similarly, PCB mixtures, driven by the enrichment of genetic information processing, clustered separately, also consistent with the PCA. Interestingly, PCB126 clustered with PCB3, which is inconsistent with physicochemical properties and morphological endpoints.

Clustering based on suppressed pathways (Figure 8D) produced a different organization from activation-based clustering for the 10 PCB compounds included in the transcriptomic analysis, indicating that pathway inhibition reflects distinct mechanisms of toxicity. Suppressed pathways were dominated by reductions in environmental information processing and metabolic systems, with clustering driven by selective transcriptional repression rather than broad stress activation. In contrast to activated pathways, 5-OH-PCB11 clustered closely with PCB mixtures, while PCB126 clustered closer to lower-chlorinated PCB metabolites. The divergence between enriched and suppressed pathway clustering highlights that activation and inhibition represent differing biological dimensions.

Comparison across clustering domains reveals three key insights. First, physicochemical similarity does not predict morphological or pathway-level toxicity. Second, morphological clustering aligns more closely with enriched pathway profiles than with physicochemical properties, indicating that pathway enrichment mediates overt developmental toxicity. Third, clustering based on suppressed pathways captures a mechanistic aspect that differs from pathway activation, reflecting selective transcriptional downregulation rather than general stress responses. These conclusions are based on qualitative comparison of clustering patterns across domains. Overall, integrating physicochemical, morphological, and pathway-level clustering offers a more comprehensive framework for hazard prediction than any single domain alone. These findings support the inclusion of metabolite-specific effects in PCB risk assessment.

## 4. Conclusions

This integrated phenotypic and transcriptomic assessment shows that the developmental toxicity of PCBs is heavily affected by metabolism and cannot be accurately determined from parent compound structure or physicochemical properties alone. Across embryonic zebrafish assays, morphological abnormalities were sensitive endpoints and frequently co-occurred with embryonic neurobehavioral disruption, indicating that larval behavioral deficits typically do not occur on their own. Hydroxylated and sulfated PCB metabolites emerged as key contributors to developmental toxicity, often exhibiting greater potency and broader endpoint coverage than their parent congeners. Importantly, individual PCB metabolites displayed distinct transcriptomic profiles rather than behaving as a grouped class, highlighting that metabolite-specific structure and substitution patterns critically shape biological responses. Similarly, PCB mixtures did not behave like individual PCB congeners; instead, two PCB mixtures showed unique phenotypic and transcriptomic signatures, emphasizing that mixture composition and interactions heavily influence developmental outcomes. These findings emphasize a critical gap in current PCB toxicology: while individual parent congeners and select metabolites have been studied extensively, the developmental toxicity of PCB metabolite mixtures remains largely unexplored. Given the co-occurrence of PCB metabolites in environmental and biological matrices, future studies of systematic evaluation of metabolite mixtures are essential for accurately characterizing real-world exposure hazards. Mechanistically, AhR activation was limited to PCB126, suggesting that most PCB- and metabolite-related developmental effects observed here occur through AhR-independent pathways. Integrative ToxPi analyses further showed that physicochemical similarity alone does not reliably predict organism-level toxicity, while convergence on shared biological pathways better correlates with developmental outcomes. Notably, PCB mixtures clustered with certain hydroxylated metabolites, emphasizing the importance of PCB metabolism in understanding mixture toxicity. Lastly, this study demonstrates that the zebrafish model is a robust, high-throughput in vivo platform for evaluating PCB toxicity. The ability to integrate morphology, behavior, transcriptomics, and pathway analyses within a single developmental framework enables effective prioritization of parent compounds, metabolites (5-OH-PCB11), and mixtures (FRM and HR-PCB mixture). Future studies integrating transcriptomic data with predictive frameworks, such as adverse outcome pathways [109], along with targeted follow-up investigations incorporating internal dosimetry and toxicokinetic modeling, may further enhance the mechanistic interpretation of PCB-induced toxicity. Overall, these findings support integrating metabolite-specific and mixture-based testing into PCB risk assessment and highlight the importance of high-throughput zebrafish screening for advancing mechanistically informed hazard identification.

## Data Availability

The original data presented in the study are openly available in the NCBI Gene Expression Omnibus at GSE326509.

## Supplementary Materials

The following supporting information can be downloaded at https://www.mdpi.com/article/10.3390/toxics14050444/s1: Synthesis of chemicals. Table S1. Abbreviations and unique identifiers of the test compounds used in this study. Table S2. Benchmark dose (BMD_10_) values that resulted in a 10% higher response (10% benchmark response) than the negative control. Values in μM. No value represents no BMD_10_ observed. Table S3. Exposure concentrations for *cyp1a* reporter analysis. Values in μM. Figure S1. Representative fluorescence images of a *cyp1a* reporter line (Tg(*cyp1a*:nls-egfp)) at 120 hpf following embryonic exposure. References [110,111,112,113,114,115,116,117,118,119,120,121,122,123,124,125,126,127] are cited in the Supplementary Materials.

## Abbreviations

The following abbreviations are used in this manuscript:

AhR	Aryl Hydrocarbon Receptor
ANOVA	Analysis of Variance
BMD10	Benchmark Dose 10%
DEGs	Differentially Expressed Genes
DL-PCBs	Dioxin-like Polychlorinated Biphenyls
DMSO	Dimethyl Sulfoxide
EGFP	Enhanced Green Fluorescence Protein
EPR	Embryonic Photomotor Response
FRM	Fox River Mixture
GEO	Gene Expression Omnibus
GO	Gene Ontology
GPCR	G protein-Coupled Receptor
GUI	Graphical User Interface
hpf	Hours Post-Fertilization
HPLC	High-Performance Liquid Chromatography
HR-PCB	Human-Relevant Polychlorinated Biphenyl
KEGG	Kyoto Encyclopedia of Genes and Genomes
LEL	Lowest Effect Level
LPR	Larval Photomotor Response
LSD	Least Significant Difference
NCBI	National Center for Biotechnology Information
NDL-PCBs	Non-Dioxin-Like Polychlorinated Biphenyls
OH-PCBs	Hydroxylated Polychlorinated Biphenyls
PCA	Principal Component Analysis
PCBs	Polychlorinated Biphenyls
RIN	RNA Integrity Number
SARL	Sinnhuber Aquatic Research Laboratory
ToxPi	Toxicological Priority Index
ZAAP	Zebrafish Acquisition and Analysis Program
