# Supplementary material for: Morphological, Behavioral, and Transcriptomic Profiling Reveals Developmental Toxicity of PCB Metabolites in Zebrafish
Source: Toxics. 2026 May 19;14(5):444. doi: 10.3390/toxics14050444 (PMC13211715; doi:10.3390/toxics14050444)
Supplement: Supplementary file 1 [file toxics-14-00444-s001.zip › toxics-4263285-supplementary.pdf]

SUPPLEMENTARY MATERIALS

Morphological, Behavioral, and Transcriptomic Profiling Reveals Developmental Toxicity of PCB Metabolites in Zebrafish

Nicole M. Breese [1,2], Lisa Truong [3], Xueshu Li [1], Robyn L. Tanguay [3] and Hans-Joachim Lehmler [1,2]

[1]Department of Occupational and Environmental Health, College of Public Health, The University of Iowa, Iowa City, Iowa 52242, USA, [2]Interdisciplinary Graduate Program in Human Toxicology, The University of Iowa, Iowa City, Iowa 52242, USA, [3]Department of Environmental and Molecular Toxicology, Oregon State University, Corvallis, OR, 97331, USA.

Corresponding Author: Dr. Hans-Joachim Lehmler
Department of Occupational and Environmental Health
The University of Iowa
University of Iowa Research Park, B164 MTF
Iowa City, IA 52242-5000
Phone: (319) 335-4310
Fax: (319) 335-4981

Table of Contents

Synthesis of Chemicals S3

Table S1. Abbreviations and unique identifiers of the test compounds used in this study. S4

Table S2. Benchmark dose (BMD_{10}) values that resulted in a 10% higher response (10% benchmark response) than the negative control. Values in μM. No value represents no BMD_{10} observed. S7

Table S3. Exposure concentrations for *cyp1a* reporter analysis. Values in μM. S9

Figure S1. Representative fluorescence images of a *cyp1a* reporter line (Tg(*cyp1a*:nls-egfp)) at 120 hpf following embryonic exposure. S10

References S11

Synthesis of Chemicals

PCB11 was synthesized via diazotization with sodium nitrite and subsequent reduction with hypophosphorous acid [1]. PCB28, PCB52, PCB95, and PCB153 were prepared and purified as described previously [2]. PCB126 was synthesized by the Suzuki coupling of 3,4,5-trichlorobromobenzene and 3,4-dichloroboronic acid [3,4]. PCB3 and 4'-OMe-PCB3 [5] were synthesized analogously via the Suzuki-coupling reaction [6,7]. PCB136 was prepared via the Ullman coupling reaction, and the PCB136 atropisomers were separated with HPLC on two serially connected Nucleodex β-PM columns [8]. Hydroxylated PCB metabolites (2'-OH-PCB3 [9], 3'-OH-PCB3 [10], 4'-OH-PCB3 [11], 4-OH-PCB11 [12], 5-OH-PCB11, 4'-OH-PCB25, and 4-OH-PCB52 [13]) were obtained by demethylation of methoxylated PCBs with BBr_3 [6,7]. 2-(4-Chlorophenyl)benzo-1,4-quinone (4-Cl-BQ) was synthesized from 1,4-benzoquinone and diazotized 4-chloroaniline using Meerwein arylation reaction [14]. The human-relevant (HR-)PCB mixture [15], Fox River PCB mixture [16], and cabinet PCB mixture [17] were prepared by mixing individual PCB congeners and/or technical PCB mixtures to approximate environmentally or human-relevant PCB profiles. PCB sulfates (3'-PCB3 sulfate, 4'-PCB3 sulfate, 4'-PCB25 sulfate, 4-PCB52 sulfate, and 4-PCB11 sulfate) were synthesized and characterized as previous described [18].

Table S1. Abbreviations and unique identifiers of the test compounds used in this study.

Abbreviation	IUPAC Name	Formula	SMILES	InChI	InChIKey	CAS Registry Number	CAS Registry URL	PubChem CID	PubChem	DTXSID	Comptox Link
PCB3	1-chloro-4-phenylbenzene	$C_{12}H_9Cl$	C1=CC=C(C=C1)C2=CC=C(C=C2)Cl	InChI=1S/C12H9Cl/c13-12-8-6-11(7-9-12)10-4-2-1-3-5-10/h1-9H	FPWNLURCHDRMHC-UHFFFAOYSA-N	2051-62-9	https://commonchemistry.cas.org/detail?cas_rn=2051-62-9	16323	https://pubchem.ncbi.nlm.nih.gov/compound/16323	DTXSID3040300	https://comptox.epa.gov/dashboard/DTXSID3040300
2’-OH-PCB3	2-(4-chlorophenyl)phenol	$C_{12}H_9ClO$	C1=CC=C(C(=C1)C2=CC=C(C=C2)Cl)O	InChI=1S/C12H9ClO/c13-10-7-5-9(6-8-10)11-3-1-2-4-12(11)14/h1-8,14H	DSSULPPMTATCMP-UHFFFAOYSA-N	64181-76-6	https://commonchemistry.cas.org/detail?cas_rn=64181-76-6	182402	https://pubchem.ncbi.nlm.nih.gov/compound/182402	DTXSID30982709	https://comptox.epa.gov/dashboard/DTXSID30982709
3’-OH-PCB3	3-(4-chlorophenyl)phenol	$C_{12}H_9ClO$	C1=CC(=CC(=C1)O)C2=CC=C(C=C2)Cl	InChI=1S/C12H9ClO/c13-11-6-4-9(5-7-11)10-2-1-3-12(14)8-10/h1-8,14H	CIPDQYWUAVNLSD-UHFFFAOYSA-N	28023-90-7	https://commonchemistry.cas.org/detail?cas_rn=28023-90-7	3015395	https://pubchem.ncbi.nlm.nih.gov/compound/3015395	DTXSID10182301	https://comptox.epa.gov/dashboard/DTXSID10182301
4′-OH-PCB3	4-(4-Chlorophenyl)phenol	$C_{12}H_9ClO$	ClC1=CC=C(C2=CC=C(O)C=C2)C=C1	InChI=1S/C12H9ClO/c13-11-5-1-9(2-6-11)10-3-7-12(14)8-4-10/h1-8,14H	ICVFJPSNAUMFCW-UHFFFAOYSA-N	28034-99-3	https://commonchemistry.cas.org/detail?cas_rn=28034-99-3	91589	https://pubchem.ncbi.nlm.nih.gov/compound/91589	DTXSID5022354	https://comptox.epa.gov/dashboard/DTXSID5022354
3’-PCB3 sulfate	3-(4-Chlorophenyl)benzenesulfonic acid	$C_{12}H_9ClO_3S$	C1=CC(=CC(=C1)S(=O)(=O)O)C2=CC=C(C=C2)Cl	InChI=1S/C12H9ClO3S/c13-11-6-4-9(5-7-11)10-2-1-3-12(8-10)17(14,15)16/h1-8H,(H,14,15,16)	UJJDOANUYGMWDF-UHFFFAOYSA-N	—	—	19985686	https://pubchem.ncbi.nlm.nih.gov/compound/19985686	—	—
4′-PCB3 sulfate	4-(4-Chlorophenyl)benzenesulfonic acid	$C_{12}H_9ClO_3S$	C1=CC(=CC=C1C2=CC=C(C=C2)Cl)S(=O)(=O)O	InChI=1S/C12H9ClO3S/c13-11-5-1-9(2-6-11)10-3-7-12(8-4-10)17(14,15)16/h1-8H,(H,14,15,16)	CPYFXXWHNKSBRR-UHFFFAOYSA-N	—	—	15429141	https://pubchem.ncbi.nlm.nih.gov/compound/15429141	—	—
4’-OMe-PCB3	1-chloro-4-(4-methoxyphenyl)benzene	$C_{13}H_{11}ClO$	COC1=CC=C(C=C1)C2=CC=C(C=C2)Cl	InChI=1S/C13H11ClO/c1-15-13-8-4-11(5-9-13)10-2-6-12(14)7-3-10/h2-9H,1H3	PUVSUQVVUYQOBK-UHFFFAOYSA-N	58970-19-7	https://commonchemistry.cas.org/detail?cas_rn=58970-19-7	612791	https://pubchem.ncbi.nlm.nih.gov/compound/612791	DTXSID20346497	https://comptox.epa.gov/dashboard/DTXSID20346497
PCB11	1-chloro-3-(3-chlorophenyl)benzene	$C_{12}H_8Cl_2$	C1=CC(=CC(=C1)Cl)C2=CC(=CC=C2)Cl	InChI=1S/C12H8Cl2/c13-11-5-1-3-9(7-	KTXUOWUHFLBZPW-	2050-67-1	https://commonchemistry.cas	16307	https://pubchem.ncbi.nlm.nih	DTXSID70872817	https://comptox.epa.gov/dash

				11)10-4-2-6-12(14)8-10/h1-8H	UHFFFAOYSA-N		.org/detail?cas_rn=2050-67-1		.gov/compound/16307		board/DTXSID70872817
4-OH-PCB11	2-chloro-4-(3-chlorophenyl)phenol	$C_{12}H_8Cl_2O$	C1=CC(=CC(=C1)Cl)C2=CC(=C(C=C2)O)Cl	InChI=1S/C12H8Cl2O/c13-10-3-1-2-8(6-10)9-4-5-12(15)11(14)7-9/h1-7,15H	JOHAARQQFBMIOV-UHFFFAOYSA-N	53890-78-1	https://commonchemistry.cas.org/detail?cas_rn=53890-78-1	186674	https://pubchem.ncbi.nlm.nih.gov/compound/186674	DTXSID10202159	https://comptox.epa.gov/dashboard/DTXSID10202159
5-OH-PCB11	3-Chloro-5-(3-chlorophenyl)phenol	$C_{12}H_8Cl_2O$	C1=CC(=CC(=C1)Cl)C2=CC(=CC(=C2)Cl)O	InChI=1S/C12H8Cl2O/c13-10-3-1-2-8(4-10)9-5-11(14)7-12(15)6-9/h1-7,15H	PCDMWQLKNQIRNA-UHFFFAOYSA-N	79881-37-1	https://commonchemistry.cas.org/detail?cas_rn=79881-37-1	190048	https://pubchem.ncbi.nlm.nih.gov/compound/190048	DTXSID10229942	https://comptox.epa.gov/dashboard/DTXSID10229942
4-OMe-PCB11	2-chloro-4-(3-chlorophenyl)-1-methoxybenzene	$C_{13}H_{10}Cl_2O$	COC1=C(C=C(C=C1)C2=CC(=CC=C2)Cl)Cl	InChI=1S/C13H10Cl2O/c1-16-13-6-5-10(8-12(13)15)9-3-2-4-11(14)7-9/h2-8H,1H3	MJFCWLSGRIKMHA-UHFFFAOYSA-N	—	—	132280042	https://pubchem.ncbi.nlm.nih.gov/compound/132280042	—	—
PCB28	2,4-dichloro-1-(4-chlorophenyl)benzene	$C_{12}H_7Cl_3$	C1=CC(=CC=C1C2=C(C=C(C=C2)Cl)Cl)Cl	InChI=1S/C12H7Cl3/c13-9-3-1-8(2-4-9)11-6-5-10(14)7-12(11)15/h1-7H	BZTYNSQSZHARAZ-UHFFFAOYSA-N	7012-37-5	https://commonchemistry.cas.org/detail?cas_rn=7012-37-5	23448	https://pubchem.ncbi.nlm.nih.gov/compound/23448	DTXSID2038310	https://comptox.epa.gov/dashboard/DTXSID2038310
4'-OH-PCB25	2-chloro-4-(2,4-dichlorophenyl)phenol	C12H7Cl3O	C1=CC(=C(C=C1C2=C(C=C(C=C2)Cl)Cl)Cl)O	InChI=1S/C12H7Cl3O/c13-8-2-3-9(10(14)6-8)7-1-4-12(16)11(15)5-7/h1-6,16H	IPQDZKABLRZERH-UHFFFAOYSA-N	358767-68-7	https://commonchemistry.cas.org/detail?cas_rn=358767-68-7	53221454	https://pubchem.ncbi.nlm.nih.gov/compound/53221454	DTXSID50686095	https://comptox.epa.gov/dashboard/chemical/details/DTXSID50686095
4′-PCB25 sulfate	2-chloro-4-(2,4-dichlorophenyl)phenyl ammonium sulfate	$C_{12}H_{10}Cl_3NO_4S$	O=S(OC(C=C1)=C(Cl)C=C1C2=C(Cl)C=C(Cl)C=C2)([O-])=O.[NH4+]	InChI=1S/C12H7Cl3O4S.H3N/...	GEYOZNBJRMQSLF-UHFFFAOYSA-N	—	—	—	—	—	—
PCB37	1,2-dichloro-4-(4-chlorophenyl)benzene	$C_{12}H_7Cl_3$	C1=CC(=CC=C1C2=CC(=C(C=C2)Cl)Cl)Cl	InChI=1S/C12H7Cl3/c13-10-4-1-8(2-5-10)9-3-6-11(14)12(15)7-9/h1-7H	YZANRISAORXTHU-UHFFFAOYSA-N	38444-90-5	https://commonchemistry.cas.org/detail?cas_rn=38444-90-5	38039	https://pubchem.ncbi.nlm.nih.gov/compound/38039	DTXSID00865914	https://comptox.epa.gov/dashboard/DTXSID00865914
PCB52	1,4-dichloro-2-(2,5-dichlorophenyl)benzene	$C_{12}H_6Cl_4$	C1=CC(=C(C=C1Cl)C2=C(C=CC(=C2)Cl)Cl)Cl	InChI=1S/C12H6Cl4/c13-7-1-3-11(15)9(5-7)10-6-8(14)2-4-	HCWZEPKLWVAEOV-UHFFFAOYSA-N	35693-99-3	https://commonchemistry.cas.org/detail?cas_rn=35693-99-3	37248	https://pubchem.ncbi.nlm.nih.gov/compound/37248	DTXSID3038305	https://comptox.epa.gov/dashboard/chemical/details/DTXSID3038305

				12(10)16/h1-6H							
4-OH-PCB52	2,5-dichloro4-(2,5-dichlorophenyl)phenol	$C12H6Cl4O$	C1=CC(=C(C=C1Cl)C2=CC(=C(C=C2Cl)O)Cl)Cl	InChI=1S/C12H6Cl4O/c13-6-1-2-9(14)7(3-6)8-4-11(16)12(17)5-10(8)15/h1-5,17H	ZKDSNFDCQYBBIU-UHFFFAOYSA -N	51274- 68-1	https://commonchemistry.cas.org/detail?cas_rn=51274-68-1	39971	https://pubchem.ncbi.nlm.nih.gov/compound/39971	DTXSID10199272	https://comptox.epa.gov/dashboard/DTXSID10199272
4-PCB52 sulfate	2,5-dichloro-4-(2,5-dichlorophenyl)phenyl ammonium sulfate	$C_{12}H_9Cl_4NO_4S$	O=S(OC(C=C1Cl)=C(Cl)C=C1C2=C(Cl)C=CC(Cl)=C2)([O-])=O.[NH4+]	InChI=1S/C12H6Cl4O4S.H3N/...	DAQCKCZRVSNHHX-UHFFFAOYSA-N	—	—	—	—	—	—
PCB95	1,2,4-trichloro-3-(2,5-dichlorophenyl)benzene	$C_{12}H_5Cl_5$	C1=CC(=C(C=C1Cl)C2=C(C=CC(=C2Cl)Cl)Cl)Cl	InChI=1S/C12H5Cl5/c13-6-1-2-8(14)7(5-6)11-9(15)3-4-10(16)12(11)17/h1-5H	GXNNLIMMEXHBKV-UHFFFAOYSA-N	38379-99-6	https://commonchemistry.cas.org/detail?cas_rn=38379-99-6	38012	https://pubchem.ncbi.nlm.nih.gov/compound/38012	DTXSID3038301	https://comptox.epa.gov/dashboard/DTXSID3038301
PCB126	1,2,3-trichloro-5-(3,4-dichlorophenyl)benzene	$C_{12}H_5Cl_5$	C1=CC(=C(C=C1C2=CC(=C(C(=C2)Cl)Cl)Cl)Cl)Cl	InChI=1S/C12H5Cl5/c13-8-2-1-6(3-9(8)14)7-4-10(15)12(17)11(16)5-7/h1-5H	REHONNLQRWTIFF-UHFFFAOYSA-N	57465-28-8	https://commonchemistry.cas.org/detail?cas_rn=57465-28-8	63090	https://pubchem.ncbi.nlm.nih.gov/compound/63090	DTXSID3032179	https://comptox.epa.gov/dashboard/DTXSID3032179
PCB136	1,2,4-trichloro-3-(2,3,6-trichlorophenyl)benzene	$C_{12}H_4Cl_6$	C1=CC(=C(C(=C1Cl)C2=C(C=CC(=C2Cl)Cl)Cl)Cl)Cl	InChI=1S/C12H4Cl6/c13-5-1-3-7(15)11(17)9(5)10-6(14)2-4-8(16)12(10)18/h1-4H	FZFUUSROAHKTTF-UHFFFAOYSA-N	38411-22-2	https://commonchemistry.cas.org/detail?cas_rn=38411-22-2	38024	https://pubchem.ncbi.nlm.nih.gov/compound/38024	DTXSID6073499	https://comptox.epa.gov/dashboard/DTXSID6073499
PCB153	1,2,4-trichloro-5-(2,4,5-trichlorophenyl)benzene	$C_{12}H_4Cl_6$	C1=C(C(=CC(=C1Cl)Cl)Cl)C2=CC(=C(C=C2Cl)Cl)Cl	InChI=1S/C12H4Cl6/c13-7-3-11(17)9(15)1-5(7)6-2-10(16)12(18)4-8(6)14/h1-4H	MVWHGTYKUMDIHL-UHFFFAOYSA-N	35065-27-1	https://commonchemistry.cas.org/detail?cas_rn=35065-27-1	37034	https://pubchem.ncbi.nlm.nih.gov/compound/37034	DTXSID2032180	https://comptox.epa.gov/dashboard/DTXSID2032180

Table S2. Benchmark dose (BMD_{10}) values that resulted in a 10% higher response (10% benchmark response) than the negative control. Values in µM. No value represents no BMD_{10} observed.

	MO24	SM24	MORT	CRAN	AXIS	EDEM	MUSC	LTRK	BRN	SKIN	NC	TCHR	ANY	All_EPR	Background_EPR	Excitatory_EPR	Refractory_EPR	All_LPR	Light_LPR	Dark_LPR
**4-PCB11 sulfate**			11.063	21.763	11.366	20.026		22.125	18.940	43.179			21.608			0.007		0.601		0.618
**PCB95**			48.136	41.531	37.597	49.368	36.265	37.619	72.807				23.054			14.647	18.794		0.007	0.168
**PCB153**					87.640								88.243	2.066		1.993			9.278	
**PCB126**			2.124	0.078	0.597		41.170	33.318	0.092	95.272			0.081			0.002				
**PCB3**			76.991											0.004						0.040
**PCB11**																24.637				
**PCB28**					69.226								55.930							
**PCB37**				79.619	56.505	45.279							65.206	6.147		1.153		0.004		0.101
**PCB52**					55.047								51.842	39.746		42.962			32.069	
**(+)-PCB136**			8.331		17.258								0.465							
**(-)-PCB136**			7.005	52.486	37.323			93.223	48.745				0.509	21.363	9.753		1.822	4.260		5.472
**2'-OH-PCB3**	33.133		21.307	8.932	9.967	0.831	8.194		16.587				20.318	5.325	9.070	5.579				
**3'-OH-PCB3**	11.263		9.354	0.942	1.454	0.716	7.906	7.083	0.712	1.933			0.896	0.469	7.007	0.625				
**4'-OH-PCB3**	15.293		0.454										0.493	5.496		1.206	0.020			
**4-OH-PCB11**	8.845		0.727	1.080	0.988	1.096	7.524	7.524	2.783				0.839	0.991		0.972				
**5-OH-PCB11**	1.225		2.670										2.084				0.067			
**4'-OH-PCB25**	5.838		0.723	2.904									2.191	0.280		0.270				
**4-OH-PCB52**	4.507		1.846										1.014				0.067			

**3'- PCB3 sulfate**			43.053	15.828	44.541	16.224	98.627	98.627	15.828				11.501				47.762			
**4'- PCB3 sulfate**			70.289	47.998	57.777	44.690	46.045	57.777	50.797				51.277				7.058			
**4'-PCB25 sulfate**			13.169		10.000								10.000	39.164		47.571				
**4- PCB52 sulfate**	26.326		46.365	0.844	17.035			49.404	31.801				46.154							
**4'-OMe-PCB3**				81.184	58.815	63.120							53.353	0.654		0.607	42.576	13.809		13.896
**4-OMe-PCB11**			0.127	43.654	43.436	49.277	78.447	29.758	63.555				42.373	1.233	45.319	1.148			6.189	
**4-Cl-BQ**			0.269										0.269							
**Cabinet Mixture**			0.040	4.321	44.705								44.935	9.850	24.235	0.001	18.752			
**Fox River Mixture (FRM)**				24.954	50.250			76.402					52.636				0.469			
**Human Relevant (HR) Mixture**				48.540	40.588			98.627					38.187			7.859				

Table S3. Exposure concentrations for *cyp1a* reporter analysis. Values in µM.

Compound	Concentration (µM)
(-)-PCB136	53.42
4-Cl-BQ	0.35
3'-OH-PCB3	7.79
4'-OH-PCB25	5.80
4'-OH-PCB3	1.83
4'-PCB25 Sulfate	15.50
4-OH-PCB11	4.33
4-OH-PCB52	1.35
5-OH-PCB11	3.26
PCB126	0.10

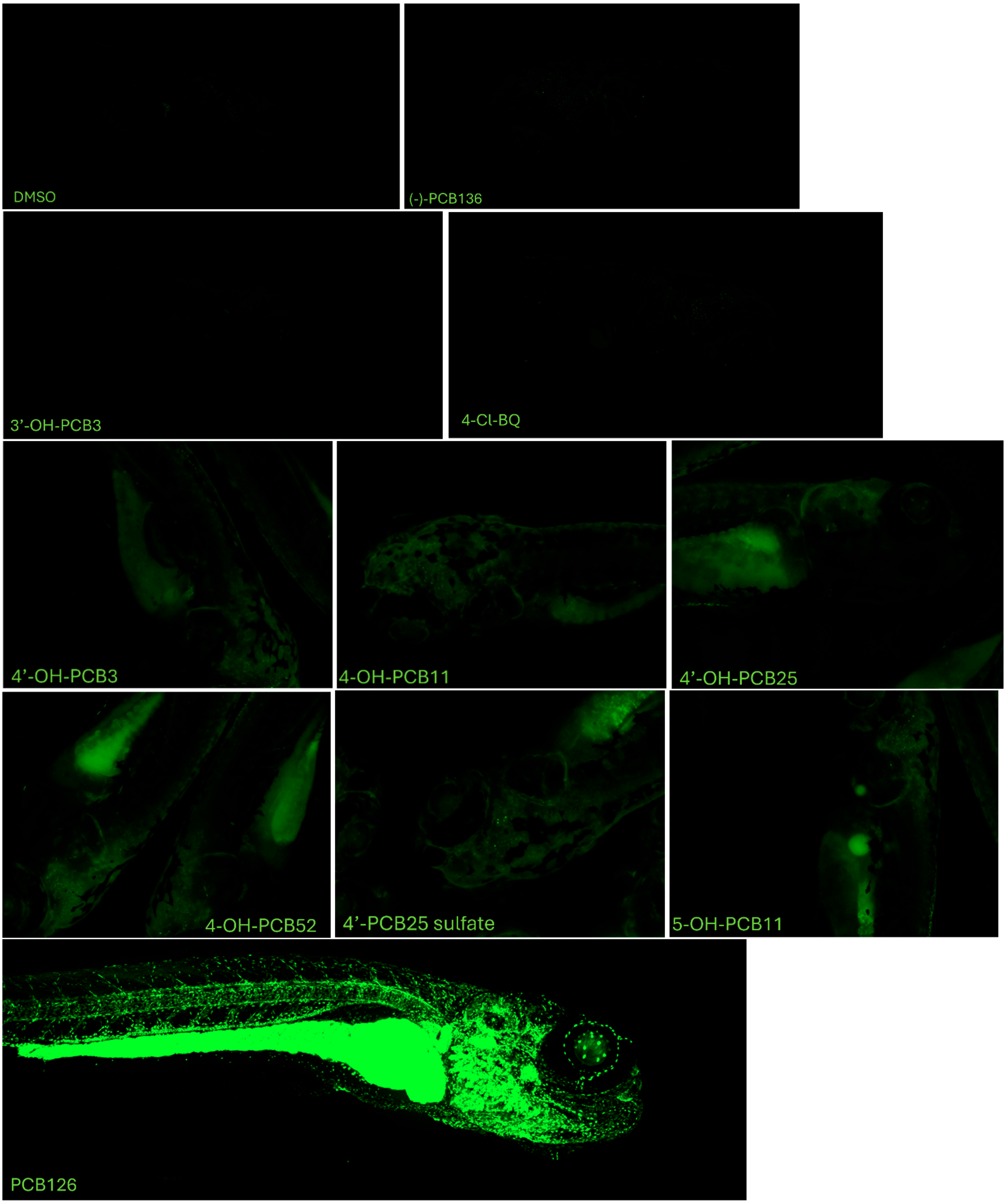

Figure S1. Representative fluorescence images of a *cyp1a* reporter line (Tg(*cyp1a*:nls-egfp)) at 120 hpf following embryonic exposure. All images captured at 10x magnification, 1/3s exposure, and max exposure = 500.

References

1. Holland, E.B.; Feng, W.; Zheng, J.; Dong, Y.; Li, X.; Lehmler, H.J.; Pessah, I.N. An extended structure-activity relationship of nondioxin-like PCBs evaluates and supports modeling predictions and identifies picomolar potency of PCB 202 towards ryanodine receptors. *Toxicol Sci* **2017**, *155*, 170–181, doi:10.1093/toxsci/kfw189.
2. Sethi, S.; Morgan, R.K.; Feng, W.; Lin, Y.; Li, X.; Luna, C.; Koch, M.; Bansal, R.; Duffel, M.W.; Puschner, B.; et al. Comparative analyses of the 12 most abundant PCB congeners detected in human maternal serum for activity at the thyroid hormone receptor and ryanodine receptor. *Environ Sci Technol* **2019**, *53*, 3948–3958, doi:10.1021/acs.est.9b00535.
3. Gadupudi, G.S.; Elser, B.A.; Sandgruber, F.A.; Li, X.; Gibson-Corley, K.N.; Robertson, L.W. PCB126 inhibits the activation of AMPK-CREB signal transduction required for energy sensing in liver. *Toxicol Sci* **2018**, *163*, 440–453, doi:10.1093/toxsci/kfy041.
4. Li, X.; Lehmler, H.-J. Authentication of 3,3',4,4',5-pentachlorobiphenyl. **2019**, doi:10.6084/m9.figshare.9736340.v1.
5. Dhakal, R.; Lehmler, H.-J. Authentication of 4-chloro-4'-methoxy-biphenyl. **2020**, doi:10.6084/m9.figshare.10299209.v1.
6. Kania-Korwel, I.; Parkin, S.; Robertson, L.W.; Lehmler, H.-J. Synthesis of polychlorinated biphenyls and their metabolites with a modified Suzuki-coupling. *Chemosphere* **2004**, *56*, 735–744, doi:10.1016/j.chemosphere.2004.04.035.
7. Shaikh, N.S.; Parkin, S.; Lehmler, H.-J. The Ullmann coupling reaction: a new approach to tetraarylstannanes. *Organometallics* **2006**, *25*, 4207–4214, doi:10.1021/om060456a.
8. Li, X.; Parkin, S.R.; Lehmler, H.J. Absolute configuration of 2,2',3,3',6-pentachlorinatedbiphenyl (PCB 84) atropisomers. *Environ Sci Pollut Res Int* **2018**, *25*, 16402–16410, doi:10.1007/s11356-017-9259-z.
9. Dhakal, R.; Lehmler, H.-J. Authentication of 4-chloro-2'-hydroxy-biphenyl. **2020**, doi:10.6084/m9.figshare.8194220.v1.
10. Dhakal, R.; Lehmler, H.-J. Authentication of 4-chloro-3'-hydroxy-biphenyl. **2020**, doi:10.6084/m9.figshare.10295243.v1.
11. Dhakal, R.; Lehmler, H.-J. Authentication of 4-chloro-4'-hydroxy-biphenyl. **2020**, doi:10.6084/m9.figshare.10299173.v1.
12. Li, X.; Lehmler, H.-J. Dataset for synthesis and authentication of 3,3'-dichlorobiphenyl-4-ol (4-OH-PCB 11). **2022**, doi:10.25820/data.006182.
13. Li, X.; Lehmler, H.-J. Dataset for synthesis and authentication of 2,2',5,5'-tetrachlorobiphenyl-4-ol (4-OH-PCB 52). **2022**, doi:10.25820/data.006178.
14. Li, X.; Lehmler, H.-J. Authentication of 2-(4'-chlorophenyl)-1,4-benzoquinone. **2019**, doi:10.6084/m9.figshare.9736379.v1.
15. Li, X.; Lehmler, H.-J. Synthesis and characterization of Alzheimer's disease and related dementias (ADRD) mixture. **2025**, doi:10.25820/data.007553.
16. Li, X.; Suh, Y.P.; Cui, J.Y.; Lehmler, H.-J. Dataset for characterization of the Fox River mixture (FRM). **2023**, doi:10.25820/data.006235.
17. Li, X.; Westra, B.; Behan-Bush, R.M.; Liszewski, J.N.; Schrodt, M.V.; Vats, B.; Klingelhutz, A.J.; Ankrum, J.A.; Lehmler, H.-J. Dataset for synthesis and characterization of Cabinet Mixture. **2022**, doi:10.25820/data.006184.
18. Li, X.; Parkin, S.; Duffel, M.W.; Robertson, L.W.; Lehmler, H.-J. An efficient approach to sulfate metabolites of polychlorinated biphenyls. *Environ Int* **2010**, *36*, 843–848, doi:10.1016/j.envint.2009.02.005.
